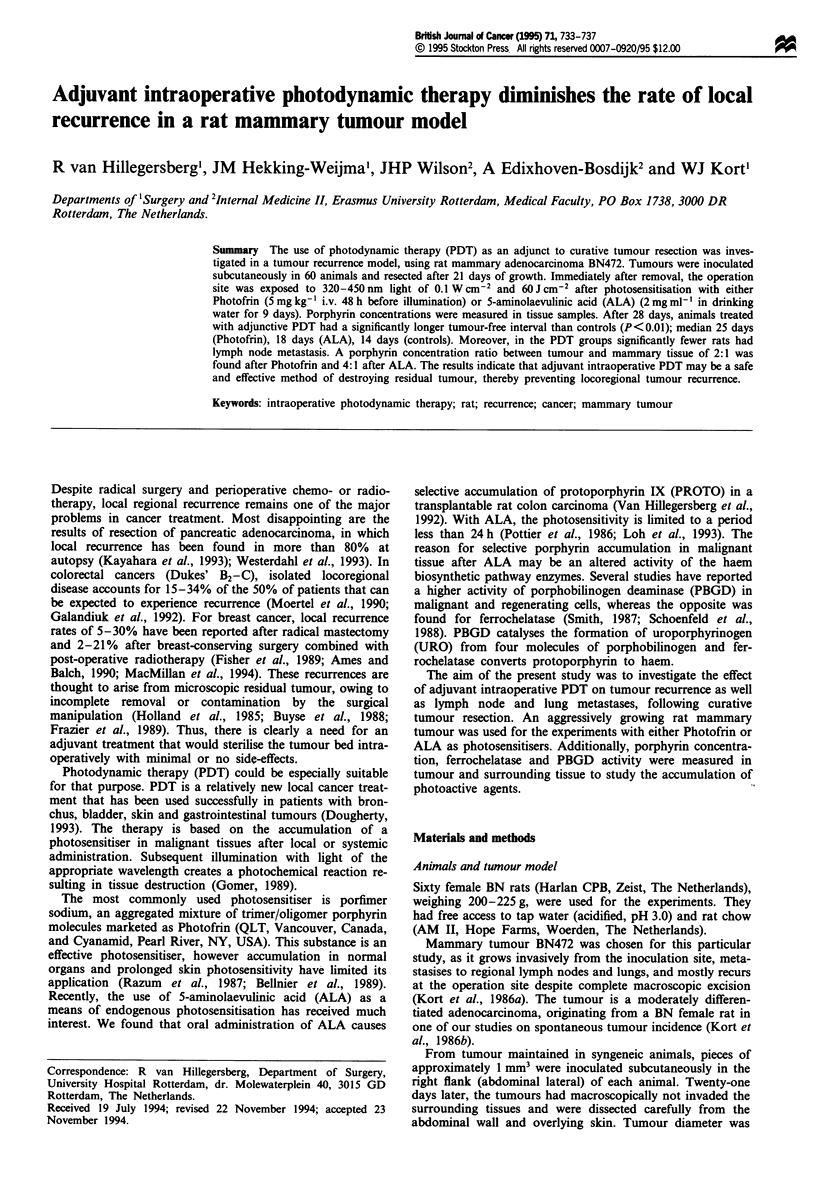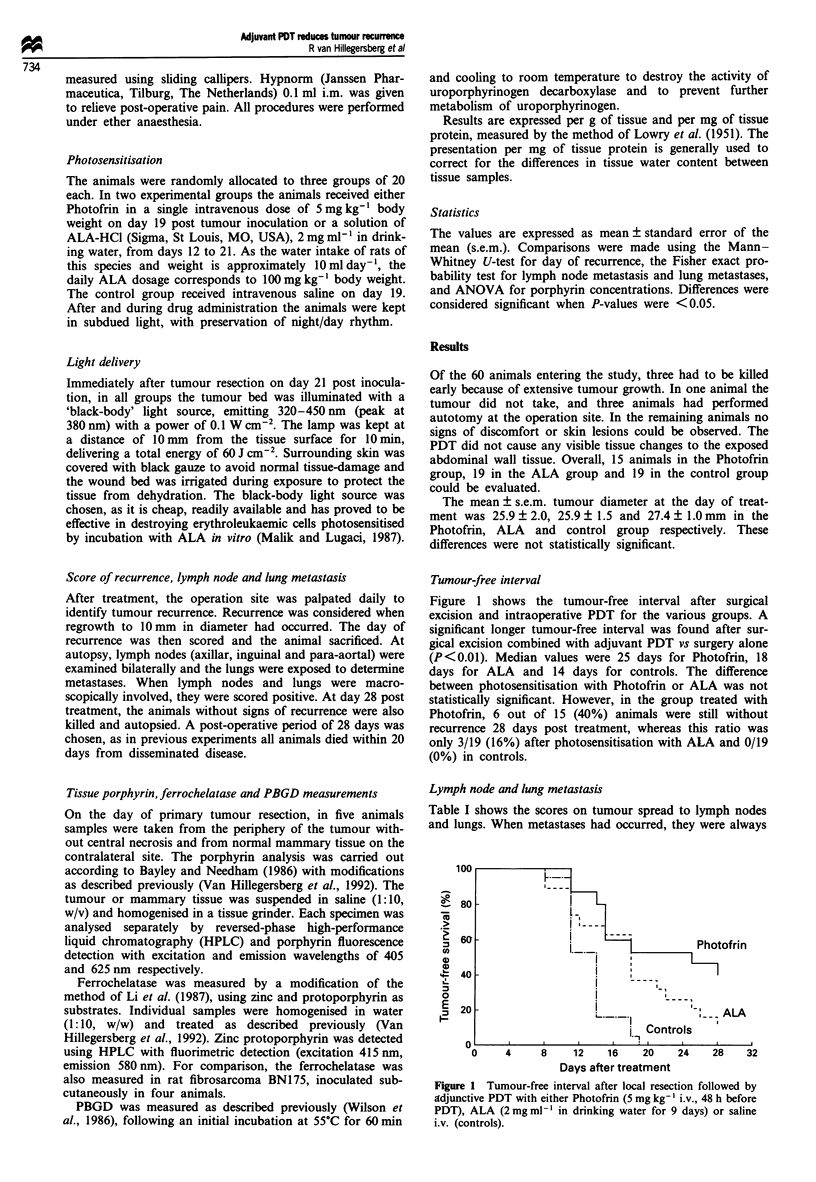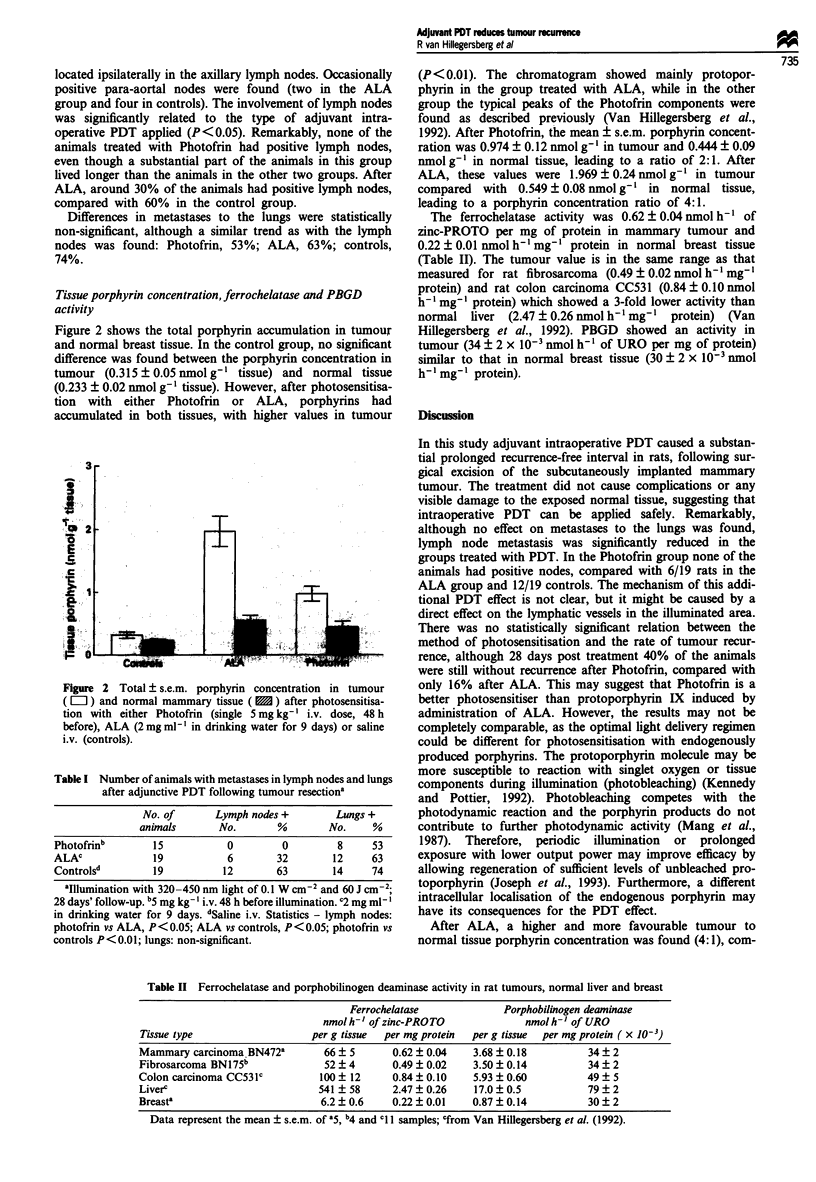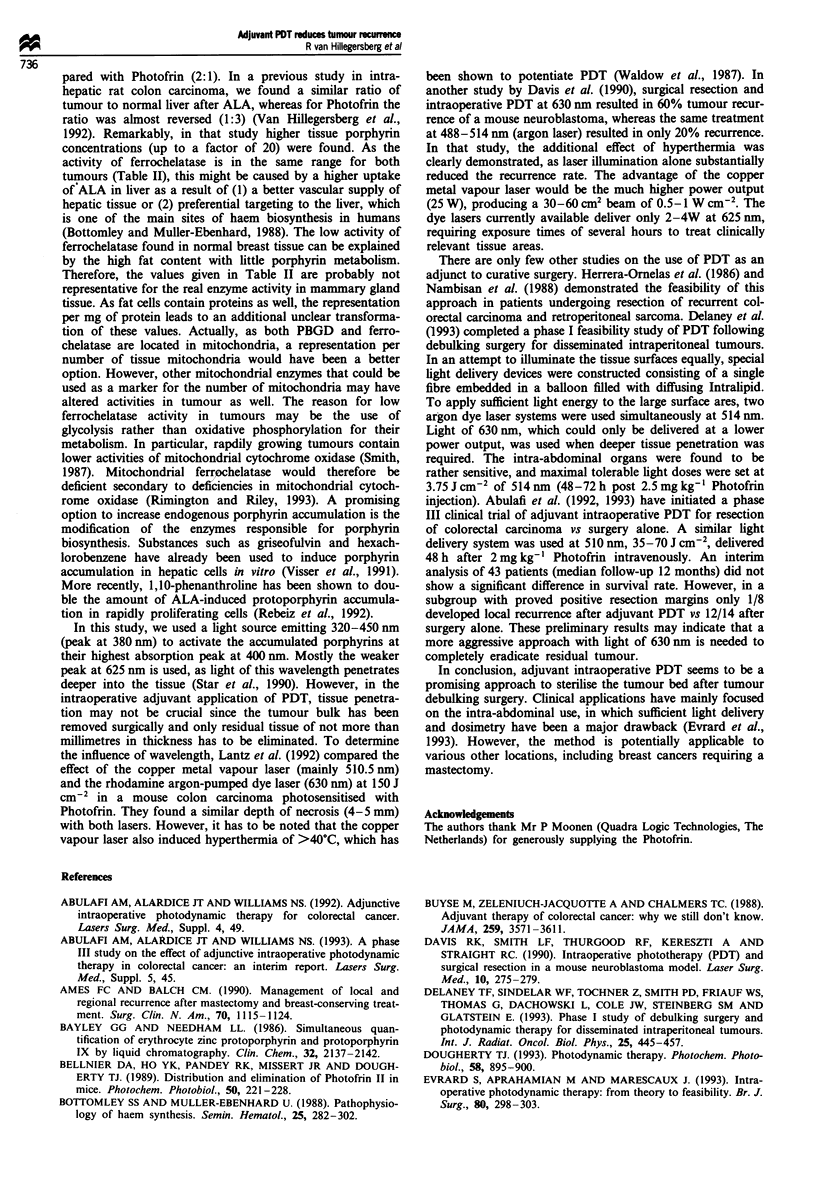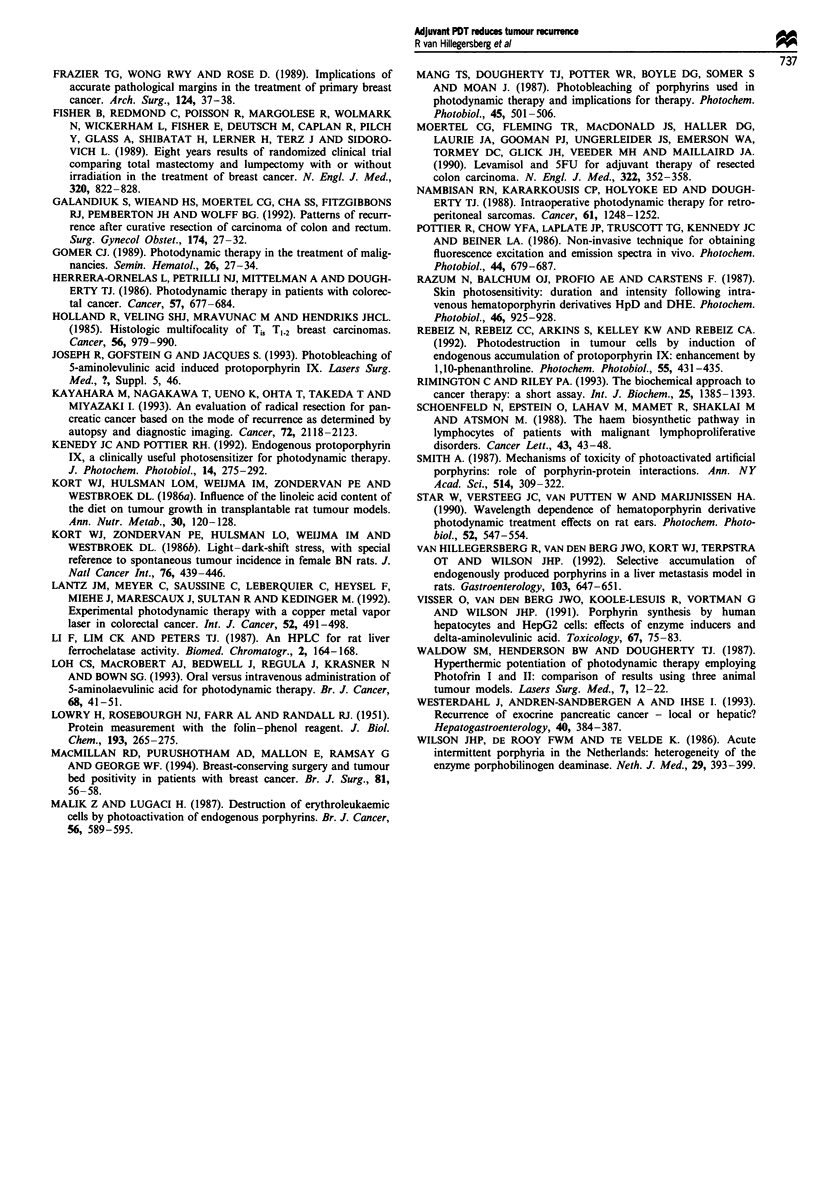# Adjuvant intraoperative photodynamic therapy diminishes the rate of local recurrence in a rat mammary tumour model.

**DOI:** 10.1038/bjc.1995.143

**Published:** 1995-04

**Authors:** R. van Hillegersberg, J. M. Hekking-Weijma, J. H. Wilson, A. Edixhoven-Bosdijk, W. J. Kort

**Affiliations:** Department of Surgery, Erasmus University Rotterdam, Medical Faculty, The Netherlands.

## Abstract

The use of photodynamic therapy (PDT) as an adjunct to curative tumour resection was investigated in a tumour recurrence model, using rat mammary adenocarcinoma BN472. Tumours were inoculated subcutaneously in 60 animals and resected after 21 days of growth. Immediately after removal, the operation site was exposed to 320-450 nm light of 0.1 W cm-2 and 60 J cm-2 after photosensitisation with either Photofrin (5 mg kg-1 i.v. 48 h before illumination) or 5-aminolaevulinic acid (ALA) (2 mg ml-1 in drinking water for 9 days). Porphyrin concentrations were measured in tissue samples. After 28 days, animals treated with adjunctive PDT had a significantly longer tumour-free interval than controls (P < 0.01); median 25 days (Photofrin), 18 days (ALA), 14 days (controls). Moreover, in the PDT groups significantly fewer rats had lymph node metastasis. A prophyrin concentration ratio between tumour and mammary tissue of 2:1 was found after Photofrin and 4:1 after ALA. The results indicate that adjuvant intraoperative PDT may be a safe and effective method of destroying residual tumour, thereby preventing locoregional tumour recurrence.


British Journal of Cancer (1995) 71, 733-737
© 1995 Stockton Press All rights reserved 0007-0920/95 $12.00

### Adjuvant intraoperative photodynamic therapy diminishes the rate of local recurrence in a rat mammary tumour model

R van Hillegersberg[1], JM Hekking-Weijma[1], JHP Wilson[2], A Edixhoven-Bosdijk[2] and WJ Kort[1]

*Departments of [1]Surgery and [2]Internal Medicine II, Erasmus University Rotterdam, Medical Faculty, PO Box 1738, 3000 DR Rotterdam, The Netherlands.*

**Summary** The use of photodynamic therapy (PDT) as an adjunct to curative tumour resection was investigated in a tumour recurrence model, using rat mammary adenocarcinoma BN472. Tumours were inoculated subcutaneously in 60 animals and resected after 21 days of growth. Immediately after removal, the operation site was exposed to 320-450 nm light of 0.1 W $cm^{-2}$ and 60 J $cm^{-2}$ after photosensitisation with either Photofrin (5 mg $kg^{-1}$ i.v. 48 h before illumination) or 5-aminolaevulinic acid (ALA) (2 mg $ml^{-1}$ in drinking water for 9 days). Porphyrin concentrations were measured in tissue samples. After 28 days, animals treated with adjunctive PDT had a significantly longer tumour-free interval than controls ($P<0.01$); median 25 days (Photofrin), 18 days (ALA), 14 days (controls). Moreover, in the PDT groups significantly fewer rats had lymph node metastasis. A porphyrin concentration ratio between tumour and mammary tissue of 2:1 was found after Photofrin and 4:1 after ALA. The results indicate that adjuvant intraoperative PDT may be a safe and effective method of destroying residual tumour, thereby preventing locoregional tumour recurrence.

**Keywords**: intraoperative photodynamic therapy; rat; recurrence; cancer; mammary tumour

Despite radical surgery and perioperative chemo- or radiotherapy, local regional recurrence remains one of the major problems in cancer treatment. Most disappointing are the results of resection of pancreatic adenocarcinoma, in which local recurrence has been found in more than 80% at autopsy (Kayahara *et al.*, 1993); Westerdahl *et al.*, 1993). In colorectal cancers (Dukes' $B_2$-C), isolated locoregional disease accounts for 15-34% of the 50% of patients that can be expected to experience recurrence (Moertel *et al.*, 1990; Galandiuk *et al.*, 1992). For breast cancer, local recurrence rates of 5-30% have been reported after radical mastectomy and 2-21% after breast-conserving surgery combined with post-operative radiotherapy (Fisher *et al.*, 1989; Ames and Balch, 1990; MacMillan *et al.*, 1994). These recurrences are thought to arise from microscopic residual tumour, owing to incomplete removal or contamination by the surgical manipulation (Holland *et al.*, 1985; Buyse *et al.*, 1988; Frazier *et al.*, 1989). Thus, there is clearly a need for an adjuvant treatment that would sterilise the tumour bed intraoperatively with minimal or no side-effects.

Photodynamic therapy (PDT) could be especially suitable for that purpose. PDT is a relatively new local cancer treatment that has been used successfully in patients with bronchus, bladder, skin and gastrointestinal tumours (Dougherty, 1993). The therapy is based on the accumulation of a photosensitiser in malignant tissues after local or systemic administration. Subsequent illumination with light of the appropriate wavelength creates a photochemical reaction resulting in tissue destruction (Gomer, 1989).

The most commonly used photosensitiser is porfimer sodium, an aggregated mixture of trimer/oligomer porphyrin molecules marketed as Photofrin (QLT, Vancouver, Canada, and Cyanamid, Pearl River, NY, USA). This substance is an effective photosensitiser, however accumulation in normal organs and prolonged skin photosensitivity have limited its application (Razum *et al.*, 1987; Bellnier *et al.*, 1989). Recently, the use of 5-aminolaevulinic acid (ALA) as a means of endogenous photosensitisation has received much interest. We found that oral administration of ALA causes selective accumulation of protoporphyrin IX (PROTO) in a transplantable rat colon carcinoma (Van Hillegersberg *et al.*, 1992). With ALA, the photosensitivity is limited to a period less than 24 h (Pottier *et al.*, 1986; Loh *et al.*, 1993). The reason for selective porphyrin accumulation in malignant tissue after ALA may be an altered activity of the haem biosynthetic pathway enzymes. Several studies have reported a higher activity of porphobilinogen deaminase (PBGD) in malignant and regenerating cells, whereas the opposite was found for ferrochelatase (Smith, 1987; Schoenfeld *et al.*, 1988). PBGD catalyses the formation of uroporphyrinogen (URO) from four molecules of porphobilinogen and ferrochelatase converts protoporphyrin to haem.

The aim of the present study was to investigate the effect of adjuvant intraoperative PDT on tumour recurrence as well as lymph node and lung metastases, following curative tumour resection. An aggressively growing rat mammary tumour was used for the experiments with either Photofrin or ALA as photosensitisers. Additionally, porphyrin concentration, ferrochelatase and PBGD activity were measured in tumour and surrounding tissue to study the accumulation of photoactive agents.

### Materials and methods

### *Animals and tumour model*

Sixty female BN rats (Harlan CPB, Zeist, The Netherlands), weighing 200-225 g, were used for the experiments. They had free access to tap water (acidified, pH 3.0) and rat chow (AM II, Hope Farms, Woerden, The Netherlands).

Mammary tumour BN472 was chosen for this particular study, as it grows invasively from the inoculation site, metastasises to regional lymph nodes and lungs, and mostly recurs at the operation site despite complete macroscopic excision (Kort *et al.*, 1986*a*). The tumour is a moderately differentiated adenocarcinoma, originating from a BN female rat in one of our studies on spontaneous tumour incidence (Kort *et al.*, 1986*b*).

From tumour maintained in syngeneic animals, pieces of approximately 1 $mm^3$ were inoculated subcutaneously in the right flank (abdominal lateral) of each animal. Twenty-one days later, the tumours had macroscopically not invaded the surrounding tissues and were dissected carefully from the abdominal wall and overlying skin. Tumour diameter was

Correspondence: R van Hillegersberg, Department of Surgery, University Hospital Rotterdam, dr. Molewaterplein 40, 3015 GD Rotterdam, The Netherlands.
Received 19 July 1994; revised 22 November 1994; accepted 23 November 1994.

measured using sliding callipers. Hypnorm (Janssen Pharmaceutica, Tilburg, The Netherlands) 0.1 ml i.m. was given to relieve post-operative pain. All procedures were performed under ether anaesthesia.

*Photosensitisation*

The animals were randomly allocated to three groups of 20 each. In two experimental groups the animals received either Photofrin in a single intravenous dose of 5 mg $kg^{-1}$ body weight on day 19 post tumour inoculation or a solution of ALA-HCl (Sigma, St Louis, MO, USA), 2 mg $ml^{-1}$ in drinking water, from days 12 to 21. As the water intake of rats of this species and weight is approximately 10 ml $day^{-1}$, the daily ALA dosage corresponds to 100 mg $kg^{-1}$ body weight. The control group received intravenous saline on day 19. After and during drug administration the animals were kept in subdued light, with preservation of night/day rhythm.

*Light delivery*

Immediately after tumour resection on day 21 post inoculation, in all groups the tumour bed was illuminated with a 'black-body' light source, emitting 320–450 nm (peak at 380 nm) with a power of 0.1 W $cm^{-2}$. The lamp was kept at a distance of 10 mm from the tissue surface for 10 min, delivering a total energy of 60 J $cm^{-2}$. Surrounding skin was covered with black gauze to avoid normal tissue-damage and the wound bed was irrigated during exposure to protect the tissue from dehydration. The black-body light source was chosen, as it is cheap, readily available and has proved to be effective in destroying erythroleukaemic cells photosensitised by incubation with ALA *in vitro* (Malik and Lugaci, 1987).

*Score of recurrence, lymph node and lung metastasis*

After treatment, the operation site was palpated daily to identify tumour recurrence. Recurrence was considered when regrowth to 10 mm in diameter had occurred. The day of recurrence was then scored and the animal sacrificed. At autopsy, lymph nodes (axillar, inguinal and para-aortal) were examined bilaterally and the lungs were exposed to determine metastases. When lymph nodes and lungs were macroscopically involved, they were scored positive. At day 28 post treatment, the animals without signs of recurrence were also killed and autopsied. A post-operative period of 28 days was chosen, as in previous experiments all animals died within 20 days from disseminated disease.

*Tissue porphyrin, ferrochelatase and PBGD measurements*

On the day of primary tumour resection, in five animals samples were taken from the periphery of the tumour without central necrosis and from normal mammary tissue on the contralateral site. The porphyrin analysis was carried out according to Bayley and Needham (1986) with modifications as described previously (Van Hillegersberg *et al.*, 1992). The tumour or mammary tissue was suspended in saline (1:10, w/v) and homogenised in a tissue grinder. Each specimen was analysed separately by reversed-phase high-performance liquid chromatography (HPLC) and porphyrin fluorescence detection with excitation and emission wavelengths of 405 and 625 nm respectively.

Ferrochelatase was measured by a modification of the method of Li *et al.* (1987), using zinc and protoporphyrin as substrates. Individual samples were homogenised in water (1:10, w/w) and treated as described previously (Van Hillegersberg *et al.*, 1992). Zinc protoporphyrin was detected using HPLC with fluorimetric detection (excitation 415 nm, emission 580 nm). For comparison, the ferrochelatase was also measured in rat fibrosarcoma BN175, inoculated subcutaneously in four animals.

PBGD was measured as described previously (Wilson *et al.*, 1986), following an initial incubation at 55°C for 60 min and cooling to room temperature to destroy the activity of uroporphyrinogen decarboxylase and to prevent further metabolism of uroporphyrinogen.

Results are expressed per g of tissue and per mg of tissue protein, measured by the method of Lowry *et al.* (1951). The presentation per mg of tissue protein is generally used to correct for the differences in tissue water content between tissue samples.

*Statistics*

The values are expressed as mean ± standard error of the mean (s.e.m.). Comparisons were made using the Mann–Whitney *U*-test for day of recurrence, the Fisher exact probability test for lymph node metastasis and lung metastases, and ANOVA for porphyrin concentrations. Differences were considered significant when *P*-values were $<0.05$.

### Results

Of the 60 animals entering the study, three had to be killed early because of extensive tumour growth. In one animal the tumour did not take, and three animals had performed autotomy at the operation site. In the remaining animals no signs of discomfort or skin lesions could be observed. The PDT did not cause any visible tissue changes to the exposed abdominal wall tissue. Overall, 15 animals in the Photofrin group, 19 in the ALA group and 19 in the control group could be evaluated.

The mean ± s.e.m. tumour diameter at the day of treatment was 25.9 ± 2.0, 25.9 ± 1.5 and 27.4 ± 1.0 mm in the Photofrin, ALA and control group respectively. These differences were not statistically significant.

*Tumour-free interval*

Figure 1 shows the tumour-free interval after surgical excision and intraoperative PDT for the various groups. A significant longer tumour-free interval was found after surgical excision combined with adjuvant PDT *vs* surgery alone ($P<0.01$). Median values were 25 days for Photofrin, 18 days for ALA and 14 days for controls. The difference between photosensitisation with Photofrin or ALA was not statistically significant. However, in the group treated with Photofrin, 6 out of 15 (40%) animals were still without recurrence 28 days post treatment, whereas this ratio was only 3/19 (16%) after photosensitisation with ALA and 0/19 (0%) in controls.

*Lymph node and lung metastasis*

Table I shows the scores on tumour spread to lymph nodes and lungs. When metastases had occurred, they were always

**Figure 1** Tumour-free interval after local resection followed by adjunctive PDT with either Photofrin (5 mg $kg^{-1}$ i.v., 48 h before PDT), ALA (2 mg $ml^{-1}$ in drinking water for 9 days) or saline i.v. (controls).

located ipsilaterally in the axillary lymph nodes. Occasionally positive para-aortal nodes were found (two in the ALA group and four in controls). The involvement of lymph nodes was significantly related to the type of adjuvant intra-operative PDT applied ($P<0.05$). Remarkably, none of the animals treated with Photofrin had positive lymph nodes, even though a substantial part of the animals in this group lived longer than the animals in the other two groups. After ALA, around 30% of the animals had positive lymph nodes, compared with 60% in the control group.

Differences in metastases to the lungs were statistically non-significant, although a similar trend as with the lymph nodes was found: Photofrin, 53%; ALA, 63%; controls, 74%.

### *Tissue porphyrin concentration, ferrochelatase and PBGD activity*

Figure 2 shows the total porphyrin accumulation in tumour and normal breast tissue. In the control group, no significant difference was found between the porphyrin concentration in tumour ($0.315 \pm 0.05$ nmol $g^{-1}$ tissue) and normal tissue ($0.233 \pm 0.02$ nmol $g^{-1}$ tissue). However, after photosensitisation with either Photofrin or ALA, porphyrins had accumulated in both tissues, with higher values in tumour

**Figure 2** Total ± s.e.m. porphyrin concentration in tumour (□) and normal mammary tissue (▨) after photosensitisation with either Photofrin (single 5 mg $kg^{-1}$ i.v. dose, 48 h before), ALA (2 mg $ml^{-1}$ in drinking water for 9 days) or saline i.v. (controls).

**Table I** Number of animals with metastases in lymph nodes and lungs after adjunctive PDT following tumour resection[a]

| | *No. of animals* | *Lymph nodes +* *No.* | *%* | *Lungs +* *No.* | *%* |
|---|---|---|---|---|---|
| Photofrin[b] | 15 | 0 | 0 | 8 | 53 |
| ALA[c] | 19 | 6 | 32 | 12 | 63 |
| Controls[d] | 19 | 12 | 63 | 14 | 74 |

[a]Illumination with 320–450 nm light of 0.1 W $cm^{-2}$ and 60 J $cm^{-2}$; 28 days' follow-up. [b]5 mg $kg^{-1}$ i.v. 48 h before illumination. [c]2 mg $ml^{-1}$ in drinking water for 9 days. [d]Saline i.v. Statistics – lymph nodes: photofrin *vs* ALA, $P<0.05$; ALA *vs* controls, $P<0.05$; photofrin *vs* controls $P<0.01$; lungs: non-significant.

($P<0.01$). The chromatogram showed mainly protoporphyrin in the group treated with ALA, while in the other group the typical peaks of the Photofrin components were found as described previously (Van Hillegersberg *et al.*, 1992). After Photofrin, the mean ± s.e.m. porphyrin concentration was $0.974 \pm 0.12$ nmol $g^{-1}$ in tumour and $0.444 \pm 0.09$ nmol $g^{-1}$ in normal tissue, leading to a ratio of 2:1. After ALA, these values were $1.969 \pm 0.24$ nmol $g^{-1}$ in tumour compared with $0.549 \pm 0.08$ nmol $g^{-1}$ in normal tissue, leading to a porphyrin concentration ratio of 4:1.

The ferrochelatase activity was $0.62 \pm 0.04$ nmol $h^{-1}$ of zinc-PROTO per mg of protein in mammary tumour and $0.22 \pm 0.01$ nmol $h^{-1}$ $mg^{-1}$ protein in normal breast tissue (Table II). The tumour value is in the same range as that measured for rat fibrosarcoma ($0.49 \pm 0.02$ nmol $h^{-1}$ $mg^{-1}$ protein) and rat colon carcinoma CC531 ($0.84 \pm 0.10$ nmol $h^{-1}$ $mg^{-1}$ protein) which showed a 3-fold lower activity than normal liver ($2.47 \pm 0.26$ nmol $h^{-1}$ $mg^{-1}$ protein) (Van Hillegersberg *et al.*, 1992). PBGD showed an activity in tumour ($34 \pm 2 \times 10^{-3}$ nmol $h^{-1}$ of URO per mg of protein) similar to that in normal breast tissue ($30 \pm 2 \times 10^{-3}$ nmol $h^{-1}$ $mg^{-1}$ protein).

### Discussion

In this study adjuvant intraoperative PDT caused a substantial prolonged recurrence-free interval in rats, following surgical excision of the subcutaneously implanted mammary tumour. The treatment did not cause complications or any visible damage to the exposed normal tissue, suggesting that intraoperative PDT can be applied safely. Remarkably, although no effect on metastases to the lungs was found, lymph node metastasis was significantly reduced in the groups treated with PDT. In the Photofrin group none of the animals had positive nodes, compared with 6/19 rats in the ALA group and 12/19 controls. The mechanism of this additional PDT effect is not clear, but it might be caused by a direct effect on the lymphatic vessels in the illuminated area. There was no statistically significant relation between the method of photosensitisation and the rate of tumour recurrence, although 28 days post treatment 40% of the animals were still without recurrence after Photofrin, compared with only 16% after ALA. This may suggest that Photofrin is a better photosensitiser than protoporphyrin IX induced by administration of ALA. However, the results may not be completely comparable, as the optimal light delivery regimen could be different for photosensitisation with endogenously produced porphyrins. The protoporphyrin molecule may be more susceptible to reaction with singlet oxygen or tissue components during illumination (photobleaching) (Kennedy and Pottier, 1992). Photobleaching competes with the photodynamic reaction and the porphyrin products do not contribute to further photodynamic activity (Mang *et al.*, 1987). Therefore, periodic illumination or prolonged exposure with lower output power may improve efficacy by allowing regeneration of sufficient levels of unbleached protoporphyrin (Joseph *et al.*, 1993). Furthermore, a different intracellular localisation of the endogenous porphyrin may have its consequences for the PDT effect.

After ALA, a higher and more favourable tumour to normal tissue porphyrin concentration was found (4:1), com-

**Table II** Ferrochelatase and porphobilinogen deaminase activity in rat tumours, normal liver and breast

| *Tissue type* | *Ferrochelatase nmol $h^{-1}$ of zinc-PROTO* *per g tissue* | *per mg protein* | *Porphobilinogen deaminase nmol $h^{-1}$ of URO* *per g tissue* | *per mg protein ( × $10^{-3}$)* |
|---|---|---|---|---|
| Mammary carcinoma BN472[a] | 66 ± 5 | 0.62 ± 0.04 | 3.68 ± 0.18 | 34 ± 2 |
| Fibrosarcoma BN175[b] | 52 ± 4 | 0.49 ± 0.02 | 3.50 ± 0.14 | 34 ± 2 |
| Colon carcinoma CC531[c] | 100 ± 12 | 0.84 ± 0.10 | 5.93 ± 0.60 | 49 ± 5 |
| Liver[c] | 541 ± 58 | 2.47 ± 0.26 | 17.0 ± 0.5 | 79 ± 2 |
| Breast[a] | 6.2 ± 0.6 | 0.22 ± 0.01 | 0.87 ± 0.14 | 30 ± 2 |

Data represent the mean ± s.e.m. of [a]5, [b]4 and [c]11 samples; [c]from Van Hillegersberg *et al.* (1992).

pared with Photofrin (2:1). In a previous study in intrahepatic rat colon carcinoma, we found a similar ratio of tumour to normal liver after ALA, whereas for Photofrin the ratio was almost reversed (1:3) (Van Hillegersberg *et al.*, 1992). Remarkably, in that study higher tissue porphyrin concentrations (up to a factor of 20) were found. As the activity of ferrochelatase is in the same range for both tumours (Table II), this might be caused by a higher uptake of ALA in liver as a result of (1) a better vascular supply of hepatic tissue or (2) preferential targeting to the liver, which is one of the main sites of haem biosynthesis in humans (Bottomley and Muller-Ebenhard, 1988). The low activity of ferrochelatase found in normal breast tissue can be explained by the high fat content with little porphyrin metabolism. Therefore, the values given in Table II are probably not representative for the real enzyme activity in mammary gland tissue. As fat cells contain proteins as well, the representation per mg of protein leads to an additional unclear transformation of these values. Actually, as both PBGD and ferrochelatase are located in mitochondria, a representation per number of tissue mitochondria would have been a better option. However, other mitochondrial enzymes that could be used as a marker for the number of mitochondria may have altered activities in tumour as well. The reason for low ferrochelatase activity in tumours may be the use of glycolysis rather than oxidative phosphorylation for their metabolism. In particular, rapdily growing tumours contain lower activities of mitochondrial cytochrome oxidase (Smith, 1987). Mitochondrial ferrochelatase would therefore be deficient secondary to deficiencies in mitochondrial cytochrome oxidase (Rimington and Riley, 1993). A promising option to increase endogenous porphyrin accumulation is the modification of the enzymes responsible for porphyrin biosynthesis. Substances such as griseofulvin and hexachlorobenzene have already been used to induce porphyrin accumulation in hepatic cells *in vitro* (Visser *et al.*, 1991). More recently, 1,10-phenanthroline has been shown to double the amount of ALA-induced protoporphyrin accumulation in rapidly proliferating cells (Rebeiz *et al.*, 1992).

In this study, we used a light source emitting 320–450 nm (peak at 380 nm) to activate the accumulated porphyrins at their highest absorption peak at 400 nm. Mostly the weaker peak at 625 nm is used, as light of this wavelength penetrates deeper into the tissue (Star *et al.*, 1990). However, in the intraoperative adjuvant application of PDT, tissue penetration may not be crucial since the tumour bulk has been removed surgically and only residual tissue of not more than millimetres in thickness has to be eliminated. To determine the influence of wavelength, Lantz *et al.* (1992) compared the effect of the copper metal vapour laser (mainly 510.5 nm) and the rhodamine argon-pumped dye laser (630 nm) at 150 J $cm^{-2}$ in a mouse colon carcinoma photosensitised with Photofrin. They found a similar depth of necrosis (4–5 mm) with both lasers. However, it has to be noted that the copper vapour laser also induced hyperthermia of $>40°C$, which has been shown to potentiate PDT (Waldow *et al.*, 1987). In another study by Davis *et al.* (1990), surgical resection and intraoperative PDT at 630 nm resulted in 60% tumour recurrence of a mouse neuroblastoma, whereas the same treatment at 488–514 nm (argon laser) resulted in only 20% recurrence. In that study, the additional effect of hyperthermia was clearly demonstrated, as laser illumination alone substantially reduced the recurrence rate. The advantage of the copper metal vapour laser would be the much higher power output (25 W), producing a 30–60 $cm^2$ beam of 0.5–1 W $cm^{-2}$. The dye lasers currently available deliver only 2–4W at 625 nm, requiring exposure times of several hours to treat clinically relevant tissue areas.

There are only few other studies on the use of PDT as an adjunct to curative surgery. Herrera-Ornelas *et al.* (1986) and Nambisan *et al.* (1988) demonstrated the feasibility of this approach in patients undergoing resection of recurrent colorectal carcinoma and retroperitoneal sarcoma. Delaney *et al.* (1993) completed a phase I feasibility study of PDT following debulking surgery for disseminated intraperitoneal tumours. In an attempt to illuminate the tissue surfaces equally, special light delivery devices were constructed consisting of a single fibre embedded in a balloon filled with diffusing Intralipid. To apply sufficient light energy to the large surface ares, two argon dye laser systems were used simultaneously at 514 nm. Light of 630 nm, which could only be delivered at a lower power output, was used when deeper tissue penetration was required. The intra-abdominal organs were found to be rather sensitive, and maximal tolerable light doses were set at 3.75 J $cm^{-2}$ of 514 nm (48–72 h post 2.5 mg $kg^{-1}$ Photofrin injection). Abulafi *et al.* (1992, 1993) have initiated a phase III clinical trial of adjuvant intraoperative PDT for resection of colorectal carcinoma *vs* surgery alone. A similar light delivery system was used at 510 nm, 35–70 J $cm^{-2}$, delivered 48 h after 2 mg $kg^{-1}$ Photofrin intravenously. An interim analysis of 43 patients (median follow-up 12 months) did not show a significant difference in survival rate. However, in a subgroup with proved positive resection margins only 1/8 developed local recurrence after adjuvant PDT *vs* 12/14 after surgery alone. These preliminary results may indicate that a more aggressive approach with light of 630 nm is needed to completely eradicate residual tumour.

In conclusion, adjuvant intraoperative PDT seems to be a promising approach to sterilise the tumour bed after tumour debulking surgery. Clinical applications have mainly focused on the intra-abdominal use, in which sufficient light delivery and dosimetry have been a major drawback (Evrard *et al.*, 1993). However, the method is potentially applicable to various other locations, including breast cancers requiring a mastectomy.

**Acknowledgements**
The authors thank Mr P Moonen (Quadra Logic Technologies, The Netherlands) for generously supplying the Photofrin.

**References**

ABULAFI AM, ALARDICE JT AND WILLIAMS NS. (1992). Adjunctive intraoperative photodynamic therapy for colorectal cancer. *Lasers Surg. Med.*, Suppl. 4, 49.

ABULAFI AM, ALARDICE JT AND WILLIAMS NS. (1993). A phase III study on the effect of adjunctive intraoperative photodynamic therapy in colorectal cancer: an interim report. *Lasers Surg. Med.*, Suppl. 5, 45.

AMES FC AND BALCH CM. (1990). Management of local and regional recurrence after mastectomy and breast-conserving treatment. *Surg. Clin. N. Am.*, **70**, 1115–1124.

BAYLEY GG AND NEEDHAM LL. (1986). Simultaneous quantification of erythrocyte zinc protoporphyrin and protoporphyrin IX by liquid chromatography. *Clin. Chem.*, **32**, 2137–2142.

BELLNIER DA, HO YK, PANDEY RK, MISSERT JR AND DOUGHERTY TJ. (1989). Distribution and elimination of Photofrin II in mice. *Photochem. Photobiol.*, **50**, 221–228.

BOTTOMLEY SS AND MULLER-EBENHARD U. (1988). Pathophysiology of haem synthesis. *Semin. Hematol.*, **25**, 282–302.

BUYSE M, ZELENIUCH-JACQUOTTE A AND CHALMERS TC. (1988). Adjuvant therapy of colorectal cancer: why we still don't know. *JAMA*, **259**, 3571–3611.

DAVIS RK, SMITH LF, THURGOOD RF, KERESZTI A AND STRAIGHT RC. (1990). Intraoperative phototherapy (PDT) and surgical resection in a mouse neuroblastoma model. *Laser Surg. Med.*, **10**, 275–279.

DELANEY TF, SINDELAR WF, TOCHNER Z, SMITH PD, FRIAUF WS, THOMAS G, DACHOWSKI L, COLE JW, STEINBERG SM AND GLATSTEIN E. (1993). Phase I study of debulking surgery and photodynamic therapy for disseminated intraperitoneal tumours. *Int. J. Radiat. Oncol. Biol. Phys.*, **25**, 445–457.

DOUGHERTY TJ. (1993). Photodynamic therapy. *Photochem. Photobiol.*, **58**, 895–900.

EVRARD S, APRAHAMIAN M AND MARESCAUX J. (1993). Intraoperative photodynamic therapy: from theory to feasibility. *Br. J. Surg.*, **80**, 298–303.

FRAZIER TG, WONG RWY AND ROSE D. (1989). Implications of accurate pathological margins in the treatment of primary breast cancer. *Arch. Surg.*, **124,** 37–38.

FISHER B, REDMOND C, POISSON R, MARGOLESE R, WOLMARK N, WICKERHAM L, FISHER E, DEUTSCH M, CAPLAN R, PILCH Y, GLASS A, SHIBATAT H, LERNER H, TERZ J AND SIDOROVICH L. (1989). Eight years results of randomized clinical trial comparing total mastectomy and lumpectomy with or without irradiation in the treatment of breast cancer. *N. Engl. J. Med.*, **320,** 822–828.

GALANDIUK S, WIEAND HS, MOERTEL CG, CHA SS, FITZGIBBONS RJ, PEMBERTON JH AND WOLFF BG. (1992). Patterns of recurrence after curative resection of carcinoma of colon and rectum. *Surg. Gynecol Obstet.*, **174,** 27–32.

GOMER CJ. (1989). Photodynamic therapy in the treatment of malignancies. *Semin. Hematol.*, **26,** 27–34.

HERRERA-ORNELAS L, PETRILLI NJ, MITTELMAN A AND DOUGHERTY TJ. (1986). Photodynamic therapy in patients with colorectal cancer. *Cancer*, **57,** 677–684.

HOLLAND R, VELING SHJ, MRAVUNAC M AND HENDRIKS JHCL. (1985). Histologic multifocality of $T_{is}$ $T_{1\text{-}2}$ breast carcinomas. *Cancer*, **56,** 979–990.

JOSEPH R, GOFSTEIN G AND JACQUES S. (1993). Photobleaching of 5-aminolevulinic acid induced protoporphyrin IX. *Lasers Surg. Med.*, **?,** Suppl. 5, 46.

KAYAHARA M, NAGAKAWA T, UENO K, OHTA T, TAKEDA T AND MIYAZAKI I. (1993). An evaluation of radical resection for pancreatic cancer based on the mode of recurrence as determined by autopsy and diagnostic imaging. *Cancer*, **72,** 2118–2123.

KENEDY JC AND POTTIER RH. (1992). Endogenous protoporphyrin IX, a clinically useful photosensitizer for photodynamic therapy. *J. Photochem. Photobiol.*, **14,** 275–292.

KORT WJ, HULSMAN LOM, WEIJMA IM, ZONDERVAN PE AND WESTBROEK DL. (1986*a*). Influence of the linoleic acid content of the diet on tumour growth in transplantable rat tumour models. *Ann. Nutr. Metab.*, **30,** 120–128.

KORT WJ, ZONDERVAN PE, HULSMAN LO, WEIJMA IM AND WESTBROEK DL. (1986*b*). Light–dark-shift stress, with special reference to spontaneous tumour incidence in female BN rats. *J. Natl Cancer Int.*, **76,** 439–446.

LANTZ JM, MEYER C, SAUSSINE C, LEBERQUIER C, HEYSEL F, MIEHE J, MARESCAUX J, SULTAN R AND KEDINGER M. (1992). Experimental photodynamic therapy with a copper metal vapor laser in colorectal cancer. *Int. J. Cancer*, **52,** 491–498.

LI F, LIM CK AND PETERS TJ. (1987). An HPLC for rat liver ferrochelatase activity. *Biomed. Chromatogr.*, **2,** 164–168.

LOH CS, MACROBERT AJ, BEDWELL J, REGULA J, KRASNER N AND BOWN SG. (1993). Oral versus intravenous administration of 5-aminolaevulinic acid for photodynamic therapy. *Br. J. Cancer*, **68,** 41–51.

LOWRY H, ROSEBOURGH NJ, FARR AL AND RANDALL RJ. (1951). Protein measurement with the folin–phenol reagent. *J. Biol. Chem.*, **193,** 265–275.

MACMILLAN RD, PURUSHOTHAM AD, MALLON E, RAMSAY G AND GEORGE WF. (1994). Breast-conserving surgery and tumour bed positivity in patients with breast cancer. *Br. J. Surg.*, **81,** 56–58.

MALIK Z AND LUGACI H. (1987). Destruction of erythroleukaemic cells by photoactivation of endogenous porphyrins. *Br. J. Cancer*, **56,** 589–595.

MANG TS, DOUGHERTY TJ, POTTER WR, BOYLE DG, SOMER S AND MOAN J. (1987). Photobleaching of porphyrins used in photodynamic therapy and implications for therapy. *Photochem. Photobiol.*, **45,** 501–506.

MOERTEL CG, FLEMING TR, MACDONALD JS, HALLER DG, LAURIE JA, GOOMAN PJ, UNGERLEIDER JS, EMERSON WA, TORMEY DC, GLICK JH, VEEDER MH AND MAILLAIRD JA. (1990). Levamisol and 5FU for adjuvant therapy of resected colon carcinoma. *N. Engl. J. Med.*, **322,** 352–358.

NAMBISAN RN, KARARKOUSIS CP, HOLYOKE ED AND DOUGHERTY TJ. (1988). Intraoperative photodynamic therapy for retroperitoneal sarcomas. *Cancer*, **61,** 1248–1252.

POTTIER R, CHOW YFA, LAPLATE JP, TRUSCOTT TG, KENNEDY JC AND BEINER LA. (1986). Non-invasive technique for obtaining fluorescence excitation and emission spectra in vivo. *Photochem. Photobiol.*, **44,** 679–687.

RAZUM N, BALCHUM OJ, PROFIO AE AND CARSTENS F. (1987). Skin photosensitivity: duration and intensity following intravenous hematoporphyrin derivatives HpD and DHE. *Photochem. Photobiol.*, **46,** 925–928.

REBEIZ N, REBEIZ CC, ARKINS S, KELLEY KW AND REBEIZ CA. (1992). Photodestruction in tumour cells by induction of endogenous accumulation of protoporphyrin IX: enhancement by 1,10-phenanthroline. *Photochem. Photobiol.*, **55,** 431–435.

RIMINGTON C AND RILEY PA. (1993). The biochemical approach to cancer therapy: a short assay. *Int. J. Biochem.*, **25,** 1385–1393.

SCHOENFELD N, EPSTEIN O, LAHAV M, MAMET R, SHAKLAI M AND ATSMON M. (1988). The haem biosynthetic pathway in lymphocytes of patients with malignant lymphoproliferative disorders. *Cancer Lett.*, **43,** 43–48.

SMITH A. (1987). Mechanisms of toxicity of photoactivated artificial porphyrins: role of porphyrin-protein interactions. *Ann. NY Acad. Sci.*, **514,** 309–322.

STAR W, VERSTEEG JC, VAN PUTTEN W AND MARIJNISSEN HA. (1990). Wavelength dependence of hematoporphyrin derivative photodynamic treatment effects on rat ears. *Photochem. Photobiol.*, **52,** 547–554.

VAN HILLEGERSBERG R, VAN DEN BERG JWO, KORT WJ, TERPSTRA OT AND WILSON JHP. (1992). Selective accumulation of endogenously produced porphyrins in a liver metastasis model in rats. *Gastroenterology*, **103,** 647–651.

VISSER O, VAN DEN BERG JWO, KOOLE-LESUIS R, VORTMAN G AND WILSON JHP. (1991). Porphyrin synthesis by human hepatocytes and HepG2 cells: effects of enzyme inducers and delta-aminolevulinic acid. *Toxicology*, **67,** 75–83.

WALDOW SM, HENDERSON BW AND DOUGHERTY TJ. (1987). Hyperthermic potentiation of photodynamic therapy employing Photofrin I and II: comparison of results using three animal tumour models. *Lasers Surg. Med.*, **7,** 12–22.

WESTERDAHL J, ANDREN-SANDBERGEN A AND IHSE I. (1993). Recurrence of exocrine pancreatic cancer – local or hepatic? *Hepatogastroenterology*, **40,** 384–387.

WILSON JHP, DE ROOY FWM AND TE VELDE K. (1986). Acute intermittent porphyria in the Netherlands: heterogeneity of the enzyme porphobilinogen deaminase. *Neth. J. Med.*, **29,** 393–399.